# Cross-Cultural Adaptation and Validation of an Attitude about Euthanasia Scale in Portuguese Older Adults with Mixed Anxiety–Depressive Disorder

**DOI:** 10.3390/healthcare12121221

**Published:** 2024-06-19

**Authors:** Luís Fonseca, Luísa Castro, Andreia Gonçalves, Francesco Monteleone, Guilhermina Rêgo, Rui Nunes

**Affiliations:** 1 Bioethics Department, Faculty of Medicine, University of Porto, 4200-319 Porto, Portugal; guilherminarego@med.up.pt (G.R.); ruinunes@med.up.pt (R.N.); 2 Psychogeriatrics Unity, Psychiatry Department, Senhora da Oliveira Hospital, 4835-044 Guimarães, Portugal; andreiamsgoncalves@gmail.com (A.G.); francescomonteleone.md@gmail.com (F.M.); 3 Department of Community Medicine, Information and Health Decisions Sciences, MEDICIS, Faculty of Medicine, University of Porto, 4200-450 Porto, Portugal; luisacastro@med.up.pt; 4 Centre for Health Technology and Services Research (CINTESIS@RISE), Faculty of Medicine, University of Porto, 4200-450 Porto, Portugal

**Keywords:** euthanasia, depression, anxiety, psychometrics, scale, older adults

## Abstract

On 25 May 2023, the Portuguese parliament approved the decriminalisation of euthanasia for incurable illnesses. As the experiences of other countries show us, it will be a matter of time before mental disorders are addressed. Studying the phenomenon, particularly in vulnerable groups, in advance is essential for proper law drafting. Therefore, instruments that allow an objective assessment and comparison between groups must be available. This study aims to explore the validation of Faria’s attitude about euthanasia scale in Portuguese older adults with mixed anxiety–depressive disorder. A sample of 114 older adults with mixed anxiety–depressive disorder collected by convenience in the Psychiatry Department of Senhora da Oliveira Hospital in Portugal was included. The pre-final version of the scale was tested in a small group with good results. The validity of the internal structure was analysed using exploratory factorial analysis. The internal consistency study verified reliability. For construct validity, we assessed the correlation with other validated scales measuring attitudes toward euthanasia, cognitive performance, personality and empathy. The attitude about euthanasia scale showed good internal consistency. One factor was retained in the principal component analysis. Significant correlations verified construct validity. The results support the scale’s usefulness and validity. This study makes available a unique instrument to assess the overall tendency of the attitudes towards euthanasia from the European-Portuguese perspective, which can be used, for example, to compare Portuguese with Brazilian older adults suffering from the same disorder. Furthermore, the adapted scale paves the way for other cross-cultural translations, adaptations, validations, and comparative analyses.

## 1. Introduction

Euthanasia has been widely discussed and progressively implemented in many countries [1,2]. Self-determination, which became the health paradigm in Western societies after World War II, is the basis for increasing acceptance [3,4,5]. Nowadays, the right of an individual to decide whether to do ancillary diagnostic tests or treatments proposed by a doctor supersedes medical authority in almost every circumstance. One of the most known exceptions to this full autonomy of patients occurs in psychiatry when, due to a severe mental disorder, the individual’s capacity to make informed decisions and give proper consent is compromised [6]. However, even here, the possibility of a person refusing treatment through an advanced healthcare directive is debated [7]. Inclusively, this has been recently considered by the Portuguese parliament in the proposal for the reform of the Mental Health Law currently in effect [8].

Regarding mental health, euthanasia is not only about whether a patient can make such a request. Many are certainly mentally competent to do so [9]. However, the quantification of “unbearable” mental suffering is much more complicated than “unbearable” physical suffering [10,11,12], and too many times, non-pharmacological treatments are not available [13]. In fact, psychopharmacology is only one of a vast array of psychiatric therapies [14]. However, a psychiatric illness is often considered refractory to treatment after only psychotropic drugs have been used [15]. This narrow interpretation of refractory illness is particularly alarming in older adults, who accumulate psychosocial needs and ageing-related health problems [16]. One systematic review of older adults’ attitudes towards euthanasia and physician-assisted suicide (PAS) found associations with some psycho and socioeconomic variables. However, it stressed that the findings were complex to compare across studies because of the variety of sample characteristics and outcome measures [17]. Hence, as far as euthanasia is concerned, it is imperative to carry out exhaustive comparative studies involving older adults with the most various psychiatric disorders to evaluate their opinion regarding euthanasia, the main reasons behind it, and the differences (if any) between this population and both healthy and physically sick individuals of other ages. Furthermore, it is essential to conduct timely and thorough evaluations of the mental capacity of older adults when considering decisions related to euthanasia. Finally, assessing whether factors other than poor medication response contribute to a reduced quality of life, lower satisfaction with health, and a specific attitude towards euthanasia must be a priority.

For this extensive and critical research, instruments that allow an objective assessment and comparison between different groups must be available. However, the existing attitude towards euthanasia scales [18,19,20] present a significant limitation. They focus on specific domains and situations (active vs. passive; doctor vs. patient decision) and, by not providing an overall opinion of a specific group of individuals about euthanasia, involve difficult cross-cultural comparisons [21].

The primary strength of the attitude about euthanasia scale (AAEs) developed by Faria [22] is to assess the overall tendency of a group of individuals regarding euthanasia and to allow intra- and intercultural comparisons. This is why its validation is essential, specifically for vulnerable groups like older adults with mental disorders. In fact, despite several articles addressing euthanasia both in older adults and mental disorders in general [23,24,25], a more objective and concise comparative analysis is lacking to fully understand the phenomenon and add information to the legalisation processes, which are currently at the centre of the discussion in a growing number of countries.

This study aims to validate AAEs in European-Portuguese older adults with mixed anxiety–depressive disorder (ICD-10).

## 2. Material and Methods

The AAEs was initially developed for Brazilian-Portuguese speakers [22]. This study’s cross-cultural adaptation of the original instrument was performed to validate the scale for European-Portuguese speakers. Then, the validation of the adapted version of the scale was pursued using exploratory factor analysis to assess the internal structure; Cronbach’s alpha, inter-item correlations and corrected item-total correlations for internal consistency; and correlation with related constructs to address construct validity.

(a) Cross-cultural adaptation

Brazilian-Portuguese native speakers developed the AAEs [22]. Some guidelines establish recommendations for the cross-cultural adaptation process [26], detailing the necessary steps for an adequate translation. However, European- and Brazilian-Portuguese speaking and writing are similar, so no translation was necessary for AAEs. Nevertheless, subtle lexicon and semantic differences exist between Brazilian-Portuguese and European-Portuguese. Thus, the questionnaire was analysed in detail by an expert committee (composed of the authors of this article, all highly proficient in European- and Brazilian-Portuguese writing and speaking) to assess if some adaptation was needed for application to Portuguese native speakers. Overall, it was concluded that the Portuguese language of the original scale was closely related to European-Portuguese writing, and no significant changes were needed. Slight differences in the verb tenses and lexicon of ten items (Table 1) were subjected to minor adjustments for an adequate reading and interpretation by the Portuguese population (Table 1). The expert committee (composed of the authors of this article: specialists in medicine, geriatric psychiatry, bioethics, statistics, and economics) reached a consensus about the pre-final version of the scale (see Supplementary Material, Table S1), which was submitted to a pre-test in 20 older adults (age ≥ 65) selected by convenience. The pre-test revealed an excellent interpretation of all the items. No doubts or objections were raised.

(b) Study design and participants

A questionnaire with a sociodemographic section and a battery of scales was applied to older adults (aged ≥ 65) with mixed anxiety–depression disorder (International Classification of Diseases, 10th Revision) with stable co-morbidities (if present). All patients were medicated with psychotropic drugs, respecting international prescribing guidelines (13th Edition of the Maudsley Prescribing Guidelines in Psychiatry [27]). Patients with depressive and anxiety symptoms secondary to non-psychiatric illness, chronic pain, and dementia were omitted. 

The study was approved by the Ethics Committee of the Senhora da Oliveira Hospital (SOH), Guimarães, Portugal (ref. 70/2020). The sample was collected in the Psychogeriatric Unit of the Psychiatry Department (PD) between 7 May 2021 and 30 November 2022. Patients who met the inclusion criteria were asked to participate voluntarily and signed an informed consent. In compliance with Portuguese law, the researchers guaranteed the participant’s data confidentiality and the right to refuse participation without any consequences. 

The PD of SOH has an area of influence of 350,000 inhabitants [28] and includes the following services: outpatient care, inpatient care, emergency service, daycare hospital, liaison psychiatry, community psychiatry, and psychogeriatric.

The number of patients included (114) aligns with the minimum reported in the literature to ensure the stability of the variance–covariance matrix in factor analysis [29].

(c) Instruments

Wasserman et al. [20] developed a 10-item scale (wATEs) to measure attitudes towards euthanasia, considering six contexts: severe pain, non-recovery, patient’s wish, physician’s authority, active euthanasia, and passive euthanasia. Participants answer using the Likert scale response categories of (1) strongly disagree, (2) disagree, (3) undecided, (4) agree, and (5) strongly agree. It delineates active and passive euthanasia, no chance for recovery and severe pain, and the patient’s autonomy and doctor’s authority. Higher scores indicate a more favourable attitude towards euthanasia in the respective domain. The Cronbach’s alpha of the original study was 0.87 [20]. The Portuguese version presented good overall internal consistency (Cronbach’s alpha = 0.90) [29], including in two separate domains, namely “Decision/Will of the Patient” (α = 0.94) and “Decision/Evaluation of the Physician” (α = 0.85), revealing a high internal consistency with values of 0.94 and 0.85, respectively [30]. 

The interpersonal reactivity index (IRI) measures empathy and includes four sub-scales: perspective taking, which reflects the tendency to adopt the other’s points of view; empathic concern, which measures the ability to experience feelings of compassion and concern for the other; personal discomfort, which assesses feelings of anxiety, apprehension, and discomfort in tense interpersonal contexts; and fantasy, which assesses the person’s propensity to place themselves in fictional situations [31]. Responses to each statement/item of the IRI are given by using a 5-level scale (“Does not describe me well” = 0; “Describes me very well” = 4; 1, 2, and 3 for intermediate answers) and the overall quotation results from the average of adding the values of each sub-scale. Higher scores indicate a higher capacity, use, or intensity of the respective dimension of empathy. The Cronbach’s’ alpha of the Portuguese version (perspective taking = 0.74; empathic concern = 0.77; personal discomfort = 0.81; fantasy = 0.83) was similar to those found with the other versions of IRI [32]. 

The NEO Five-Factor Inventory (NEO-FFI) measures five personality factors (neuroticism, extraversion, openness, agreeableness, and conscientiousness), with 12 items for each factor [33]. Each of the items is measured on a Likert-based scale ranging from 0 (“Strongly Disagree”) to 4 (“Strongly Agree”). Higher scores in each domain indicate a higher impact of that particular personality trait. The Portuguese version of NEO-FFI revealed good reliability with Cronbach’s alpha values (conscientiousness = 0.81, neuroticism = 0.81, extraversion = 0.75, agreeableness = 0.72, and openness = 0.71) related to those reported for the original NEO-FFI [34]. 

Mini-mental-state (MMS) [35,36] and clock-drawing test (CDT) [37,38] evaluate one’s cognitive ability, which is closely related to education level [39,40]. In the MMS, [35,36] Portuguese scores are as follows: possible cognitive decline if MMS ≤ 22 for subjects from 1 to 11 years of education, ≤27 for those with 11 years of education, and ≤15 for illiterate. The CDT uses a 10-point quantitative system encompassing three major clock components (circle picturing, numbers writing, and clock hands drawing). In our study, ten past eleven (11:10) was used as a reference, as recommended by several authors [41,42] Scores ≤ 6 were considered abnormal [37,38,40].

(d) Statistical analysis

Analyses were performed using the statistical software package SPSS Statistics (v. 28.0; SPSS^®^ Inc., Chicago, IL, USA). The categorical variables were described by absolute and relative frequencies, n (%). The quantitative variables presented deviations from the normal distribution and were described by median and interquartile interval: Med (1stQ, 3rdQ). The normality of quantitative variables was evaluated by visual inspection of the respective histograms. Correlations were calculated using the Spearman correlation coefficient, *r*. Values of *p* ≤ 0.05 were considered significant.

Internal structure, reliability, and construct validity were analysed to validate the aAAEs for Portuguese older adults.

An exploratory factor analysis was performed to access the scale’s internal structure, following the procedure adopted in the original article [22]. First, and to assess the suitability of the sample for use in the analysis, both the Keiser–Meyer–Olkin (KMO) measurement and Bartlett’s sphericity test were conducted. Then, factor extraction was performed using the principal component analysis method with Varimax (orthogonal) rotation. Kaiser’s method (keep factors with eigenvalues greater than 1), the percentage of explained variance higher than 5%, and the interpretation of the scree plot flattening point were considered to decide the number of factors to retain. The results are reported regarding factor loadings for each item and item communalities, with the latter describing the proportion of the item’s variance explained by the factor(s) retained [43].

The scale’s reliability was studied through internal consistency, measured by Cronbach’s alpha (α) [44] and McDonald’s omega (ω) coefficients. Cronbach’s alpha measures the interrelation between items of a one-dimensional scale or subscale, and values between 0.7 and 0.9 are acceptable [45,46]. Cronbach’s alpha relies on a measurement model that assumes that the indicators are essentially tau-equivalent; that is, the relationship between items and the measured construct is assumed to be constant across the items (among other conditions). However, this is often not the case. In fact, it is more of an exception than a rule. Research indicates that alpha tends to estimate reliability less accurately than omega when tau-equivalence is not met. Hence, MacDonalds’ omega has emerged as a recommended alternative, and in this study, both were computed. The mean of the correlations between items should be between 0.15 and 0.5 to ensure they assess the same construct and are not redundant (Clark and Watson, 1995). Each item must correlate with the total of the construct (item-total corrected correlation) with values between 0.3 and 0.7 [45]. Effects of “ceiling” or “floor” are present when more than 15% of respondents reach the theoretical maximum or minimum of the scale, respectively. Therefore, these effects limit the scale’s validity and should be verified [47].

Several studies found a more favourable attitude towards euthanasia in individuals with better empathic skills [48,49], higher educational levels [50,51] and some personality traits [17,50,52], like openness to experience and agreeableness. Thus, to test these hypotheses in our sample and to verify the construct validity [44] of the aAAEs, correlations were calculated between aAAEs and the IRI [31,32], which measures empathy; the NEO-FFI [33,34], which assesses the emotional, behavioural, and cognitive tendencies of the individuals in five significant categories of personality traits; and MMS [35,36] and CDT [37,38] to evaluate education-related cognitive performance [39,40]. Furthermore, the correlation with wATEs [20], already validated for the Portuguese population [30], was also tested to verify the equivalence of measures.

Hospital anxiety and depression scale (HADS) [53,54], MARS therapy adherence scale (TAS) [55,56], and Barthel index (BI) [57,58] were also used to assess depression and anxiety, therapeutic adherence, and level of independence for daily life activities, respectively.

## 3. Results

One hundred and fourteen patients medicated with antidepressants participated in the study (Table 2). The group consisted mainly of women (76.3%), and the mean age was 73 (average ± SD, min-max: 73.01 ± 5.32, 65–91) years old. Most were married (60.5%), and 76.3% of the household included two or more people. Additionally, 81.6% had four years of schooling or less. Most were Catholic (97.4%) and retired (98.2%). Psychometric assessment (Table 3) revealed that most patients presented mild depressive and anxiety symptoms (HADS), normal cognitive performance (as shown by MMS and CDT scores), and good therapeutic adherence (MARS) and were fully independent for daily life activities (BI).

All the scales used presented high internal consistency:

- wATEs—α = 0.98, ω = 0.97; “Patient’s Decision/Will”: α = 0.97, ω = 0.97; “Doctor’s Decision/Evaluation”: α = 0.98, ω = 0.98;
- IRI—perspective taking: α = 0.94, ω = 0.94; empathic concern: α = 0.84, ω = 0.85; personal discomfort: α = 0.92, ω = 0.91; fantasy: α = 0.89, ω = 0.90;
- NEO-FFI—conscientiousness: α = 0.84, ω = 0.84; neuroticism: α = 0.93, ω = 0.93; extraversion: α = 0.86, ω = 0.87; agreeableness: α = 0.62, ω = 0.64; openness: α = 0.86, ω = 0.88.

Significant and positive correlations were found between schooling/MMS and schooling/CDT (Schooling/MMS: r(112) = 0.48 (*p* < 0.001); Schooling/CDT: r(112) = 0.56 (*p* < 0.001)), which is in line with previous findings that cognitive abilities are closely related to educational level.

## 4. Validation of aAAEs

(a) Validation of the internal structure

Data revealed good adequacy to a factorial analysis through the measure KMO = 0.96, having rejected the hypothesis of correlation matrix equal to identity by Bartlett’s test of sphericity (df = 325; *p* < 0.001).

After extracting the factors, it was found that only two resulted in an eigenvalue greater than 1. The first factor alone explains about 72% of the total data variability, and the second explains 4.5%. Since the second factor accounted for less than 5% of the explained variance, retaining only the first factor is appropriate. The visual analysis of the scree plot corroborates this. As two of the three criteria support the retention of only one factor, we propose this factorisation (Table 4). The commonalities for the 26 observed variables ranged from 0.47 to 0.89, with a mean value of 0.72. The variable with the highest commonality was item 19 (0.89), which suggests it is a strong indicator of the construct being measured. The variable with the lowest communality was item 1 (0.48), which may suggest that it is less strongly related to the underlying factor. However, all commonalities were within an acceptable range and provided evidence of good measurement quality for the observed variables.

Data indicated adequate internal consistency of the aAAEs total scale: a Cronbach’s alpha of 0.98, a MacDonald’s omega of 0.98, a mean item-item correlation of 2.7 (ranging from 1.91 to 3.15), and corrected item-total correlations between 0.67 and 0.94 and no “floor” or “ceiling” effects.

(b) Construct validity

The original studies of AAEs focused only on the association between the attitude toward euthanasia and religious beliefs or the attitude towards euthanasia of certain professional groups, specifically doctors and lawyers [59]. Later studies have found relationships between empathic skills [48,49], personality [17,50,52], and educational level [50,51] and the individuals’ attitude towards euthanasia.

In this study, significant positive correlations of aAAEs were found with both dimensions of wATEs—“Patient’s Decision/Will” (r(112) = 0.90; *p* < 0.001) and “Doctor’s Decision/Evaluation” (r(112) = 0.83; *p* < 0.001); the IRI perspective taking (r(112) = 0.76; *p* < 0.001), empathic concern ((r(112) = 0.32; *p* < 0.001) and fantasy [r(112) = 0.38; *p* < 0.001) domains; and the MMS (r(112) = 0.29; *p* = 0.002) and CDT (r(112) = 0.25; *p* = 0.007) and with the NEO-FFI openness to experience (r(112) = 0.23; *p* = 0.013), agreeableness (r(112) = 0.22; *p* = 0.017), and conscientiousness (r(112) = 0.29; *p* = 0.002) domains. Likewise, a significant negative correlation of AAEs was found with the IRI personal discomfort (r(112) = −0.20; *p* = 0.033) domain.

These results indicate the instrument’s construct validity in this group of patients.

## 5. Discussion

On 25 May 2023, the Portuguese parliament decriminalised euthanasia [60]. Self-determined adults can request it in a situation of great suffering and with definitive injury of extreme severity or incurable illness (“life-threatening disease”). Furthermore, medically assisted death can only occur by euthanasia when medically assisted suicide is impossible due to the patient’s physical incapacity. The law neither lists any diseases nor explicitly excludes psychiatric disorders from euthanasia. Nevertheless, as mentioned earlier, mental disorders present specific problems related to the subjectivity of unbearable suffering and the daily clinical consideration of refractory disease. This is particularly troublesome in older adults, where ageing-related issues, illiteracy, and psychosocial frailties are prominent. Some Portuguese older adults with four years of schooling or less have minimal reading or writing skills, with some only knowing how to sign their name and many unable to perform “simple” digital tasks like handling a smartphone or texting [61]. In addition, people lose much of their autonomy when they grow old and fragile, being increasingly inclined or forced to leave decisions to others [62], which may compromise free and informed decisions. Thus, research addressing potential circumstances and factors that might influence attitudes towards euthanasia is of the utmost importance, particularly in older adults. In Portugal, depressive and anxiety disorders appear in the sixth and ninth position, respectively, of the ten top causes of disability-adjusted life years [63], and the estimated prevalence of anxiety among older adults is 9.6%, while depression is 11.8% [64]. Therefore, we prioritise addressing these disorders, especially when Portugal faces a dramatic demographic winter [65]. Several studies have been trying to understand how empathy [48,49], personality traits [17,50,52], education [50,51], or loneliness [51,66,67] affect one’s judgment of unbearable suffering and attitudes toward euthanasia. However, the variety of the methodology used and the absence of similar measure’ tools hinder the comparison between studies and compromise a better understanding of the phenomenon. AAEs [22], which objectively assesses a group of individuals’ opinions regarding euthanasia and allows cross-cultural comparisons, is a psychometric tool that can aid in overcoming those limitations. Its use in comparative analysis will help to clarify differences regarding the attitudes towards euthanasia, allowing for tailored legislation and health and public policies to be implemented. Also, understanding how the overall attitude towards euthanasia relates to psychiatric disorders severity, suicidal ideation, psychosocial factors, personality traits, educational level, and ageing-related issues will contribute to targeted prevention and treatment strategies, enhancing people’s autonomy, well-being, and quality of life.

The complete form of the original AAEs was validated with eight samples and used in research [22,68] with highly satisfactory results. In our study, aAAEs proved to be a valid and reliable instrument for evaluating the attitude toward euthanasia in older adults with mixed anxiety–depressive disorder, with a good representation of all items by the single extracted factor (in the unifactorial solution, the commonality varied between 0.47 and 0.89).

Regarding construct validity, statistically significant correlations were found between aAAEs and IRI, some NEO-FFI domains, and MMS and CDT, indicating that a more favourable tendency towards euthanasia is associated with higher perspective taking, empathic concern, and fantasy capacities; lower personal discomfort; personality traits of openness to experience, agreeableness, and conscientiousness; and higher education. These results align with several findings from other studies where the same variables (empathy [48,49], personality [17,50,52], and education [50,51]) were considered. Furthermore, the significant positive correlation between aAAEs and both dimensions of wATEs, that is, between two different scales that measure the same variable, adds value to construct validity and suggests equivalence of measures [43,69].

Our research not only makes cross-culturally adapted and validated AAEs available, allowing for immediate comparisons between Brazilian and Portuguese older adults with a mixed anxiety–depressive disorder, but it also contributes to characterising its factor structure. 

Some limitations should be noticed in our study. Firstly, because the participants were selected by convenience (older adult patients of the Psychogeriatric Department), obeying the inclusion criteria, the sample may not be representative of the elderly Portuguese population. Secondly, although the number of participants followed the recommended minimum for such studies [29], large samples are advised for reducing measurement errors and producing generalisable results for the population. Finally, the consistency of AAEs across time (test–retest reliability) still needs to be explored. This can be particularly interesting in clinical practice when following-up with patients. Thus, future works should address its temporal consistency. 

Novel strategies such as complex dynamic systems theory (CDST), an emerging research approach, can be considered in future studies. It provides formulas for examining non-linear, multifaceted elements, behaviours, or phenomena (e.g., euthanasia) and allows the identification of factors that influence them [70]. In fact, the CDST framework has proven to be appropriate for analysing factors that may be in a state of flux and change with even subtle variations of contexts, as can happen in the attitude towards euthanasia.

## 6. Conclusions

The AAEs presented good internal consistency and construct validity, and the exploratory factor analysis proposes a one-factor structure of the instrument. Hence, its use in research should be extended to several age groups and diseases. Furthermore, additional transcultural validations should be encouraged. A better understanding of the attitude towards euthanasia in several settings and cultures will allow the implementation of tailored legislation and health strategies.

## Figures and Tables

**Table 1:** Items adjusted to European-Portuguese writing.

Item Number	Original Items of AAEs	Adjusted Items of aAAEs
1	É um absurdo manter uma pessoa com vida vegetativa através de aparelhagem artificial. (It is absurd to maintain a person in a vegetative life through artificial devices.)	É um absurdo manter uma pessoa em vida vegetativa através de aparelhos artificiais. (It is absurd to maintain a person in a vegetative life through artificial devices.)
2	Se existe condição de manter vivo um doente com recursos artificiais, mesmo por tempo indefinido, não se deve praticar a eutanásia. (If there are conditions to keep a patient alive with artificial resources, even for an indefinite time, euthanasia should not be practiced.)	Se existem condições para manter vivo um doente com recursos artificiais, mesmo que por tempo indefinido, não se deve praticar a eutanásia. (If there are conditions to keep a patient alive with artificial resources, even for an indefinite time, euthanasia should not be practiced.)
3	Não hesitaria em abreviar, sem dor ou sofrimento, a vida de um doente reconhecidamente condenado. (I would not hesitate to abbreviate, without pain or suffering, the life of an admittedly condemned patient.)	Não hesitaria em abreviar, sem dor nem sofrimento, a vida de um doente reconhecidamente condenado. (I would not hesitate to abbreviate, without pain or suffering, the life of an admittedly condemned patient.)
7	Não me sentiria culpado permitindo a eutanásia em um de meus familiares que estivesse desenganado e sofrendo terrivelmente. (I would not feel guilty allowing the euthanasia of one of my family members with hopeless and terrible suffering.)	Não me sentiria culpado permitindo a eutanásia de um dos meus familiares que estivesse desenganado e a sofrer terrivelmente. (I would not feel guilty allowing the euthanasia of one of my family members with hopeless and terrible suffering.)
10	Deve-se manter o doente incurável, o tempo necessário, apenas com paliativos em vez de apressar-lhe a morte. (The incurable patient should be maintained, for as long as necessary, only with palliative care instead of abbreviating their death.)	Deve-se manter o doente incurável, o tempo que for preciso, apenas com paliativos em vez de lhe abreviar a morte. (The incurable patient should be maintained, for as long as necessary, only with palliative care instead of abbreviating their death.)
14	Aquele que pensa saber a hora certa para alguém deixar de viver se julga Todo-Poderoso. (Those who think knowing the right time for someone to stop living think they are Almighty.)	Aquele que pensa saber a hora certa para alguém deixar de viver julga-se Todo-Poderoso. (Those who think knowing the right time for someone to stop living think they are Almighty.)
17	Há certas circunstâncias em que a pessoa tem todo o direito de decidir se quer ou não continuar esperando a morte. (There are certain circumstances in which a person has every right to decide whether to continue waiting for death.)	Há certas circunstâncias em que a pessoa tem todo o direito de decidir se quer ou não continuar à espera da morte. (There are certain circumstances in which a person has every right to decide whether to continue waiting for death.)
24	Quem autoriza a morte de um parente, na verdade, está querendo aliviar sua própria dor. (Those who authorize the death of a relative truly want to alleviate their own pain.)	Quem autoriza a morte de um parente, na verdade, quer aliviar a sua própria dor. (Those who authorize the death of a relative truly want to alleviate their own pain.)
25	Se o desligamento da aparelhagem artificial não resolver, deve ser aplicada uma injeção que adiante a morte do condenado. (If turning off the artificial life support machines does not solve the problem, an injection must be applied to anticipate the death of the condemned patient.)	Se desligar as máquinas de suporte artificial à vida não resolver, deve ser aplicada uma injeção que adiante a morte do condenado. (If turning off the artificial life support machines does not solve the problem, an injection must be applied to anticipate the death of the condemned patient.)
26	O sofrimento do doente incurável faz parte de sua missão na Terra, por isso a eutanásia não deve ser praticada. (The suffering of the incurably ill is part of their mission on Earth, so euthanasia should not be practiced.)	O sofrimento do doente incurável faz parte da sua missão na Terra, por isso a eutanásia não deve ser praticada. (The suffering of the incurably ill is part of their mission on Earth, so euthanasia should not be practiced.)

AAEs, attitude about euthanasia scale; aAAEs, adapted attitude about euthanasia scale.

**Table 2:** Sociodemographic descriptive analysis (N = 114).

Variable	Descriptive
Gender, n (%)	
Woman	87 (76.3)
Man	27 (23.7)
Age (years), average ± SD, min-max	73.01 ± 5.32, 65–91
Schooling years, n (%)	
0	7 (6.1)
1	2 (1.8)
2	1 (0.9)
3	13 (11.4)
4	70 (61.4)
6	5 (4.4)
7	1 (0.9)
8	1 (0.9)
9	3 (2.6)
11	2 (1.8)
12	4 (3.5)
15	2 (1.8)
16	1 (0.9)
17	2 (1.8)
Grouped schooling (for MMS), n (%)	
Illiterate (0 years)	7 (6.1)
1 to 11 years	98 (86)
>11 years	9 (7.9)
Civil status, n (%)	
Married	69 (60.5)
Divorced	12 (10.5)
Single	7 (6.1)
Widow	26 (22.8)
Religion, n (%)	
Catholic	111 (97.4)
Jehovah’s witness	2 (1.8)
Agnostic	1 (0.9)
Profession, n (%)	
Self-employed	1 (0.9)
Manager	1 (0.9)
Retired	112 (98.2)
Household size, n (%)	
1	27 (23.7)
2	51 (44.7)
3	25 (21.9)
4	7 (6.1)
5	2 (1.8)
6	1 (0.9)
11	1 (0.9)

MMS, mini-mental-state.

**Table 3:** Descriptive analysis of the scales (N = 114).

Scales and Subscales	Descriptive
HADS Depression, med (IQI), min–max	9 (6; 10), 0–15
HADS Depression, n (%)	
0–7: asymptomatic	34 (29.8)
8–10: mild depression	67 (58.8)
11–14: moderate depression	11 (9.6)
15–21: severe depression	2 (1.8)
HADS anxiety, med (IQI), min–max	8 (5; 10), 1–19
HADS anxiety, n (%)	
0–7: asymptomatic	35 (30.7)
8–10: mild anxiety	54 (47.4)
11–14: moderate anxiety	22 (19.3)
15–21: severe anxiety	3 (2.6)
MMS, med (IQI), min–max	28 (26; 29), 11–30
MMS (without cognitive deficit), n (%) MMS (with cognitive deficit:), n (%)	109 (95.6)
Illiterate ≤ 15	1 (0.9)
1 to 11 years of schooling ≤ 22	4 (3.5)
Schooling years higher than 11 ≤ 27	0 (0)
CDT, med (IQI), min–max	9 (7; 9.5), 1–10
CDT, n (%)	
Normal (>6)	89 (78.1)
Cognitive deficit (≤6)	25 (21.9)
MARS, med (IQI), min–max	9 (7; 9), 0–10
MARS, n (%)	
No adherence: <4	1 (0.9)
Partial adherence: 4 to 7	28 (24.6)
Good adherence: >7	85 (74.6)
BI, med (IQI), min–max	100 (100; 100), 70–100
BI, n (%)	
0–20: total dependence	0 (0)
21–60: severe dependence	0 (0)
61–90: moderate dependence	6 (5.3)
91–99: very mild dependence	10 (8.8)
100: full independence	98 (86)

Note: SD, standard deviation; min, minimum; max, maximum; med, median; IQI, interquartile interval (1stQ; 3rdQ); HADS, hospital anxiety and depression scale; MMS, mini-mental-state; CDT, clock drawing test; MARS, medication adherence rating scale; BI, Barthel index.

**Table 4:** Factor loadings of the 26 items of the adapted attitude about euthanasia scale (aAAEs) in the one-factor solution extracted by the principal components’ method (N = 114).

aAAEs Item	Factor Loading
1. É um absurdo manter uma pessoa em vida vegetativa através de aparelhos artificiais. (It is absurd to maintain a person in a vegetative life through artificial devices.)	0.69
2. Se existem condições para manter vivo um doente com recursos artificiais, mesmo que por tempo indefinido, não se deve praticar a eutanásia. (If there are conditions to keep a patient alive with artificial resources, even for an indefinite time, euthanasia should not be practiced.)	0.85
3. Não hesitaria em abreviar, sem dor nem sofrimento, a vida de um doente reconhecidamente condenado. (I would not hesitate to abbreviate, without pain or suffering, the life of an admittedly condemned patient.)	0.73
4. A prática da eutanásia é uma violência camuflada. (The practice of euthanasia is a camouflaged violence.)	0.85
5. A eutanásia é um direito do Homem. (Euthanasia is a right of Humankind.)	0.88
6. O Homem não deve ter o direito de abreviar a vida de outro, nem para poupá-lo de sofrimentos maiores que o levarão irremediavelmente à morte. (Humankind should not have the right to abbreviate the life of another, not even to spare them from greater suffering that will irremediably lead to death.)	0.81
7. Não me sentiria culpado permitindo a eutanásia de um dos meus familiares que estivesse desenganado e a sofrer terrivelmente. (I would not feel guilty allowing the euthanasia of one of my family members with hopeless and terrible suffering.)	0.80
8. É cobardia optar pela eutanásia. (It is cowardice to choose euthanasia.)	0.80
9. A eutanásia deve ser praticada em situações definidas, simplesmente, por razões humanitárias. (Euthanasia should be practiced in defined situations, simply for humanitarian reasons.)	0.86
10. Deve-se manter o doente incurável, o tempo que for preciso, apenas com paliativos em vez de lhe abreviar a morte. (The incurable patient should be maintained, for as long as necessary, only with palliative care instead of abbreviating their death.)	0.88
11. Abreviar com a morte os sofrimentos de alguém que se ama é, antes de tudo, um ato de humanidade. (Abbreviating the suffering of someone you love with death is, above all, an act of humanity.)	0.93
12. A família que realmente ama o doente nunca autoriza a eutanásia. (The family that really loves the patient never authorizes euthanasia.)	0.82
13. Uma legislação especial permitindo a prática a eutanásia seria uma conquista da humanidade. (A special legislation allowing the practice of euthanasia would be an achievement of humanity.)	0.90
14. Aquele que pensa saber a hora certa para alguém deixar de viver julga-se Todo-Poderoso. (Those who think knowing the right time for someone to stop living think they are Almighty.)	0.82
15. Se o doente não está mais lúcido e a família assumir a decisão, a eutanásia deve ser praticada. (If the patient is no longer lucid and the family assumes the decision, euthanasia should be practiced.)	0.84
16. Por motivos vários não deve haver uma legislação especial permitindo a prática da eutanásia. (For various reasons, there should be no special legislation allowing the practice of euthanasia.)	0.86
17. Há certas circunstâncias em que a pessoa tem todo o direito de decidir se quer ou não continuar à espera da morte. (There are certain circumstances in which a person has every right to decide whether to continue waiting for death.)	0.84
18. O direito de vida e o direito de morte escapam, sob qualquer circunstância, ao âmbito de decisão do Ser Humano. (The right to life and the right to death escape, under any circumstances, the scope of decision of the Human Being.)	0.89
19. Ter direito à eutanásia é ter direito a uma morte digna. (Having the right to euthanasia is having the right to a dignified death.)	0.94
20. Praticar a eutanásia é matar por amor, mas não deixa de ser assassinato. (Practicing euthanasia is killing for love, but it is still murder.)	0.89
21. É um crime manter um ser humano em vida puramente vegetativa. (Keeping a human being in a purely vegetative life is a crime.)	0.74
22. A eutanásia não se aplica a nenhum caso, pois cada doente reage de forma diferente a uma mesma doença. (Euthanasia does not apply to any case, as each patient reacts differently to the same disease.)	0.90
23. Uma legislação especial permitindo a prática da eutanásia deveria ser ampla, abrangendo o maior número de casos incuráveis e fatais. (Special legislation allowing the practice of euthanasia should be broad, covering the greatest number of incurable and fatal cases.)	0.91
24. Quem autoriza a morte de um parente, na verdade. quer aliviar a sua própria dor. (Whoever authorize the death of a relative truly want to alleviate their own pain.)	0.75
25. Se desligar as máquinas de suporte artificial à vida não resolver, deve ser aplicada uma injeção que adiante a morte do condenado. (If turning off the artificial life support machines does not solve the problem, an injection must be applied to anticipate the death of the condemned patient.)	0.89
26. O sofrimento do doente incurável faz parte da sua missão na Terra, por isso a eutanásia não deve ser praticada. (The suffering of the incurably ill is part of their mission on Earth, so euthanasia should not be practiced.)	0.90

## Data Availability

Data are contained within the article and Supplementary Materials.

## Supplementary Materials

The following supporting information can be downloaded at: https://www.mdpi.com/article/10.3390/healthcare12121221/s1, Table S1: Complete pre-final version of attitude about euthanasia scale.

## References

[1] Dyer O., White C., Rada A.G. (2015). Assisted dying: Law and practice around the world. BMJ.

[2] Steck N., Egger M., Maessen M., Reisch T., Zwahlen M. (2013). Euthanasia and assisted suicide in selected European countries and US states. Med. Care.

[3] Katz J. (1996). The Nuremberg Code and the Nuremberg Trial. JAMA.

[4] Will J.F. (2011). A brief historical and theoretical perspective on patient autonomy and medical decision-making, Part I: The Beneficence Model. Chest.

[5] Will J.F. (2011). A brief historical and theoretical perspective on patient autonomy and medical decision-making, Part II: The Autonomy Model. Chest.

[6] Van Staden C.W. (2003). Incapacity to give informed consent owing to mental disorder. J. Med. Ethics.

[7] Khazaal Y., Manghi R., Delahaye M., Machado A., Penzenstadler L., Molodynski A. (2014). Psychiatric advance directives, a possible way to overcome coercion and promote empowerment. Front. Public Health.

[8] Lei da Saúde Mental Act 35/2023, 21 July (Portugal). https://www.pgdlisboa.pt/leis/lei_mostra_articulado.php?nid=3679&tabela=leis&ficha=1&pagina=1&so_miolo=.

[9] Calcedo-Barba A., Fructuoso A., Martínez-Raga J., Paz S., De Carmona M.S., Vicens E. (2020). A meta-review of literature reviews assessing the capacity of patients with severe mental disorders to make decisions about their healthcare. BMC Psychiatry.

[10] Dees M., Vernooij-Dassen M.J.F.J., Dekkers W.J.M., Van Weel C. (2010). Unbearable suffering of patients with a request for euthanasia or physician-assisted suicide: An integrative review. Psycho-Oncol..

[11] Trachsel M., Jox R.J. (2022). Suffering is not enough: Assisted dying for people with mental illness. Bioethics.

[12] Verhofstadt M., Thienpont L., Peters G.J. (2017). When unbearable suffering incites psychiatric patients to request euthanasia: Qualitative study. Br. J. Psychiatry.

[13] Lake J., Turner M.S. (2017). Urgent need for improved mental health care and a more collaborative model of care. Perm. J..

[14] Carvajal C. (2004). Poor response to treatment: Beyond medication. Dialogues Clin. Neurosci..

[15] Howes O., Thase M.E., Pillinger T. (2021). Treatment resistance in psychiatry: State of the art and new directions. Mol. Psychiatry.

[16] McKee K., Schüz B. (2015). Psychosocial factors in healthy ageing. Psychol. Health.

[17] Aghababaei N., Wasserman J.A., Hatami J. (2013). Personality Factors and Attitudes toward Euthanasia in Iran: Implications for End-of-Life Research and Practice. Death Stud..

[18] Holloway H.D., Hayslip B., Murdock M.E., Maloy R., Servaty H.L., Henard K., Lopez L., Lysaght R., Moreno G., Moroney T. (1995). Measuring Attitudes toward Euthanasia. OMEGA-J. Death Dying.

[19] Tordella M.A., Neutens J.J. (1979). An instrument to appraise attitudes of college students toward euthanasia. J. Sch. Health.

[20] Wasserman J.A., Clair J.M., Ritchey F.J. (2005). A Scale to Assess Attitudes toward Euthanasia. OMEGA-J. Death Dying.

[21] Aghababaei N., Farahani H., Hatami J. (2011). Euthanasia attitude; A comparison of two scales. J. Med. Ethics Hist. Med..

[22] Faria Y.S. (1988). Escala de Atitude Sobre Eutanásia. https://bibliotecadigital.fgv.br/dspace/handle/10438/28192.

[23] De Leo D., Spathonis K. (2003). Suicide and euthanasia in late life. Aging Clin. Exp. Res..

[24] López-Castromán J. (2017). About the practice of psychiatric euthanasia: A commentary. BMC Med..

[25] Death with Dignity and Mental Disorder Arizona Law Review. https://arizonalawreview.org/death-with-dignity-and-mental-disorder/.

[26] Beaton D., Bombardier C., Guillemin F., Ferraz M.B. (2000). Guidelines for the process of Cross-Cultural adaptation of Self-Report measures. Spine.

[27] Taylor D., Barnes T., Young A. (2018). The Maudsley Prescribing Guidelines in Psychiatry.

[28] Gil N., Cintra P. História dos Serviços de Saúde Mental, 1st ed.; Autores e Edições Parsifal; 2016. https://bibliografia.bnportugal.gov.pt/bnp/bnp.exe/registo?1954037.

[29] Taherdoost H., Sahibuddin S., Jalaliyoon N. (2014). Exploratory Factor Analysis; Concepts and Theory. Appl. Pure Math..

[30] Rosa C., Gouveia M. (2015). Atitudes Perante a Eutanásia e Ansiedade Perante a Morte Numa Amostra de Estudantes Universitários. Unpublished. Master’s Thesis.

[31] Davis M. (1980). A Multidimensional Approach to Individual Differences in Empathy. J. Personal. Soc. Psychol..

[32] Limpo T., Alves R., Castro S. (2010). Medir a empatia: Adaptação portuguesa do Índice de Reactividade Interpessoal. Laboratório Psicol..

[33] Costa P.T., McCrae R.R. (2008). The Revised NEO Personality Inventory (NEO-PI-R).

[34] Magalhães E., Salgueira A.P., Gonzalez A.J., Costa J.J., Costa M.J., Costa P., de Lima M.P. (2014). NEO-FFI: Psychometric properties of a short personality inventory in Portuguese context. Psicol. Reflexão E Crítica.

[35] Folstein M.F., Folstein S.E., McHugh P.R. (1975). Mini-mental state. J. Psychiatr. Res..

[36] Guerreiro M. (1998). Contributo da Neuropsicologia para o Estudo das Demências. Unpublished. Doctoral Dissertation.

[37] Cacho J., García-García R., Arcaya J., Vicente J.L., Lantada N. (1999). Una propuesta de aplicación y puntuación del test del reloj en la enfermedad de Alzheimer [A proposal for application and scoring of the Clock Drawing Test in Alzheimer’s disease]. Rev. De Neurol..

[38] Rouleau I., Salmon D.P., Butters N., Kennedy C.M., McGuire K. (1992). Quantitative and qualitative analyses of clock drawings in Alzheimer’s and Huntington’s disease. Brain Cogn..

[39] Santana I., Duro D., Lemos R., Costa V., Pereira M., Simões M.R., Freitas S. (2016). Mini-Mental State Examination: Avaliação dos Novos Dados Normativos no Rastreio e Diagnóstico do Défice Cognitivo. Acta Médica Port..

[40] Santana I., Duro D., Freitas S., Alves L.B., Simões M.R. (2013). The Clock Drawing Test: Portuguese norms, by age and education, for three different scoring systems. Arch. Clin. Neuropsychol..

[41] Goodglass H., Kaplan E. (1972). The Assessment of aphasia and Related Disorders.

[42] Kaplan EBull T., Bryant B.K. (1988). A process approach to neuropsychological assessment. Clinical Neuropsychology and Brain Function: Research, Measurement, and Practice.

[43] Marôco J. Análise Estatística com o SPSS Statistics, 8th ed.; Report Number; 2021. https://bibliografia.bnportugal.gov.pt/bnp/bnp.exe/registo?2068652.

[44] Mokkink L.B., Terwee C.B., Patrick D.L., Alonso J., Stratford P.W., Knol D.L., Bouter L.M., de Vet H.C. (2010). The COSMIN study reached international consensus on taxonomy, terminology, and definitions of measurement properties for health-related patient-reported outcomes. J. Clin. Epidemiol..

[45] Streiner D.L., Norman G.R. (2008). Biases in responding. Oxford University Press eBooks.

[46] Hundleby J.D. (1968). Reviews: Nunnally, Jum. Psychometric Theory. New York: McGraw-Hill, 1967. 640 + xiii pp. $12.95. Am. Educ. Res. J..

[47] Terwee C.B., Bot S.D., de Boer M.R., van der Windt D.A., Knol D.L., Dekker J., Bouter L.M., de Vet H.C. (2007). Quality criteria were proposed for measurement properties of health status questionnaires. J. Clin. Epidemiol..

[48] Avci E. (2022). The goals of Medicine and Compassion in the ethical Assessment of Euthanasia and Physician-Assisted Suicide: Relieving pain and suffering by protecting, promoting, and maintaining the person’s Well-Being. J. Palliat. Care.

[49] Van Tol D.G., Rietjens J., Van Der Heide A. (2012). Empathy and the application of the ‘unbearable suffering’ criterion in Dutch euthanasia practice. Health Policy.

[50] Castelli Dransart D.A., Lapierre S., Erlangsen A., Canetto S.S., Heisel M., Draper B., Lindner R., Richard-Devantoy S., Cheung G., Scocco P. (2019). A systematic review of older adults’ request for or attitude toward euthanasia or assisted-suicide. Aging Ment. Health.

[51] Buiting H.M., Deeg D.J., Knol D.L., Ziegelmann J.P., Pasman H.R., Widdershoven G.A., Onwuteaka-Philipsen B.D. (2012). Older people’s attitudes towards euthanasia and an end-of-life pill in The Netherlands: 2001–2009. J. Med. Ethics.

[52] Wasserman J.A., Aghababaei N., Nannini D. (2015). Culture, personality, and attitudes toward euthanasia. OMEGA-J. Death Dying.

[53] Pais-Ribeiro J., Silva I., Ferreira T., Martins T., Meneses R.F., Baltar M. (2007). Validation study of a Portuguese version of the Hospital Anxiety and Depression Scale. Psychol. Health Med..

[54] Zigmond A.S., Snaith R.P. (1983). The hospital anxiety and Depression scale. Acta Psychiatr. Scand..

[55] Thompson K., Kulkarni J., Sergejew A. (2000). Reliability and validity of a new Medication Adherence Rating Scale (MARS) for the psychoses. Schizophr. Res..

[56] Vanelli I., Chendo I., Góis C., Santos J., Levy P. (2011). Adaptação e validação da versão portuguesa da escala de adesão à terapêutica. Acta Médica Port..

[57] Mahoney F.I., Barthel D.W. (1965). Functional evaluation: The barthel index. Md. State Med. J..

[58] Araújo F., Ribeiro J., Oliveira A., Pinto C. (2005). Validação do Índice de Barthel numa amostra de idosos não institucionalizados. Rev. Port. Saúde Pública.

[59] Faria Y. (1989). Atitude de médicos e advogados em relação à Eutanásia. Arq. Bras. Psicol..

[60] Condições em que a morte medicamente assistida não é punível Act 22/2023, 25 May (Portugal). https://www.pgdlisboa.pt/leis/lei_mostra_articulado.php?nid=3648&tabela=leis&ficha=1&pagina=1&so_miolo=.

[61] Fonseca L., Monteleone F., Gonçalves A., Rêgo G., Nunes R. (2023). Decision-Making Capacity of Elderly Patients with Mixed Depression-Anxiety Disorder. Acta Médica Port..

[62] Van Der Geest S., Satalkar P.P. (2019). Autonomy and dying: Notes about decision-making and “completed life” euthanasia in the Netherlands. Death Stud..

[63] Global Health Estimates: Leading Causes of DALYs. https://www.who.int/data/gho/data/themes/mortality-and-global-health-estimates/global-health-estimates-leading-causes-of-dalys.

[64] de Sousa R.D., Rodrigues A.M., Gregório M.J., Branco J.D.C., Gouveia M.J., Canhão H., Dias S.S. (2017). Anxiety and depression in the Portuguese older adults: Prevalence and associated factors. Front. Med..

[65] Projeções de População Residente (2020). Statistics Portugal. https://www.ine.pt/xportal/xmain?xpid=INE&xpgid=ine_destaques&DESTAQUESdest_boui=406534255&DESTAQUESmodo=2&xlang=pt.

[66] Ettema E.J., Derksen L.D., Van Leeuwen E. (2010). Existential loneliness and end-of-life care: A systematic review. Theor. Med. Bioeth..

[67] Van Baarsen B. (2009). Suffering, Loneliness, and the Euthanasia Choice: An Explorative study. J. Soc. Work End—Life Palliat. Care.

[68] Faria Y. (1986). Relação Entre Atitude Sobre Eutanásia e Crenças Religiosas; Col:8875|com:8874. https://bibliotecadigital.fgv.br/dspace/handle/10438/8928.

[69] Frongillo E.A., Baranowski T., Subar A.F., Tooze J.A., Kirkpatrick S.I. (2019). Establishing Validity and Cross-Context Equivalence of Measures and Indicators. J. Acad. Nutr. Diet..

[70] Derakhshan A., Wang Y., Wang Y., Ortega-Mart J. (2023). Towards innovative research approaches to investigating the role of emotional variables in promoting language teachers’ and learners’ mental health. Int. J. Ment. Health Promot..

