# Supplementary material for: Cross-Cultural Adaptation and Validation of an Attitude about Euthanasia Scale in Portuguese Older Adults with Mixed Anxiety–Depressive Disorder
Source: Healthcare (Basel). 2024 Jun 19;12(12):1221. doi: 10.3390/healthcare12121221 (PMC11204183; doi:10.3390/healthcare12121221)
Supplement: Supplementary file 1 [file healthcare-12-01221-s001.zip › healthcare-3050426-supplementary.pdf]

Table S1.

ITEMS ADJUSTED to EUROPEAN-PORTUGUESE WRITING

Item number	Original items of AAEs	Adjusted and final items of the aAAEs
**1**	É um absurdo manter uma pessoa com vida vegetativa através de aparelhagem artificial. *(It is absurd to maintain a person in a vegetative life through artificial devices.)*	É um absurdo manter uma pessoa em vida vegetativa através de aparelhos artificiais. *(It is absurd to maintain a person in a vegetative life through artificial devices.)*
**2**	Se existe condição de manter vivo um doente com recursos artificiais, mesmo por tempo indefinido, não se deve praticar a eutanásia. (*If there are conditions to keep a patient alive with artificial resources, even for an indefinite time, euthanasia should not be practiced.)*	Se existem condições para manter vivo um doente com recursos artificiais, mesmo que por tempo indefinido, não se deve praticar a eutanásia. (*If there are conditions to keep a patient alive with artificial resources, even for an indefinite time, euthanasia should not be practiced.)*
**3**	Não hesitaria em abreviar, sem dor ou sofrimento, a vida de um doente reconhecidamente condenado. *(I would not hesitate to abbreviate, without pain or suffering, the life of an admittedly condemned patient.)*	Não hesitaria em abreviar, sem dor nem sofrimento, a vida de um doente reconhecidamente condenado. *(I would not hesitate to abbreviate, without pain or suffering, the life of an admittedly condemned patient.)*
**4**	A prática da eutanásia é uma violência camuflada. *(The practice of euthanasia is a camouflaged violence.)*	A prática da eutanásia é uma violência camuflada. *(The practice of euthanasia is a camouflaged violence.)*
**5**	A eutanásia é um direito do Homem. *(Euthanasia is a right of Humankind.)*	A eutanásia é um direito do Homem. *(Euthanasia is a right of Humankind.)*
**6**	O Homem não deve ter o direito de abreviar a vida de outro, nem para poupá-lo de sofrimentos maiores que o levarão irremediavelmente à morte. *(Humankind should not have the right to abbreviate the life of another, not even to spare them from greater suffering that will irremediably lead to death.)*	O Homem não deve ter o direito de abreviar a vida de outro, nem para poupá-lo de sofrimentos maiores que o levarão irremediavelmente à morte. *(Humankind should not have the right to abbreviate the life of another, not even to spare them from greater suffering that will irremediably lead to death.)*
**7**	Não me sentiria culpado permitindo a eutanásia em um de meus familiares que estivesse desenganado e sofrendo terrivelmente. *(I would not feel guilty allowing the euthanasia of one of my family members with hopeless and terrible suffering.)*	Não me sentiria culpado permitindo a eutanásia de um dos meus familiares que estivesse desenganado e a sofrer terrivelmente. *(I would not feel guilty allowing the euthanasia of one of my family members with hopeless and terrible suffering.)*
**8**	É cobardia optar pela eutanásia. *(It is cowardice to choose euthanasia.)*	É cobardia optar pela eutanásia. *(It is cowardice to choose euthanasia.)*

Item number	Original items of AAEs	Adjusted and final items of the aAAEs
**9**	A eutanásia deve ser praticada em situações definidas, simplesmente, por razões humanitárias. *(Euthanasia should be practiced in defined situations, simply for humanitarian reasons.)*	A eutanásia deve ser praticada em situações definidas, simplesmente, por razões humanitárias. *(Euthanasia should be practiced in defined situations, simply for humanitarian reasons.)*
**10**	Deve-se manter o doente incurável, o tempo necessário, apenas com paliativos em vez de apressar-lhe a morte. *(The incurable patient should be maintained for as long as necessary, only with palliative care instead of abbreviating their death.)*	Deve-se manter o doente incurável, o tempo que for preciso, apenas com paliativos em vez de lhe abreviar a morte. *(The incurable patient should be maintained, for as long as necessary, only with palliative care instead of abbreviating their death.)*
**11**	Abreviar com a morte os sofrimentos de alguém que se ama é, antes de tudo, um ato de humanidade. *(Abbreviating the suffering of someone you love with death is, above all, an act of humanity.)*	Abreviar com a morte os sofrimentos de alguém que se ama é, antes de tudo, um ato de humanidade. *(Abbreviating the suffering of someone you love with death is, above all, an act of humanity.)*
**12**	A família que realmente ama o doente nunca autoriza a eutanásia. *(The family that really loves the patient never authorizes euthanasia.)*	A família que realmente ama o doente nunca autoriza a eutanásia. *(The family that really loves the patient never authorizes euthanasia.)*
**13**	Uma legislação especial permitindo a prática a eutanásia seria uma conquista da humanidade. *(A special legislation allowing the practice of euthanasia would be an achievement of humanity.)*	Uma legislação especial permitindo a prática a eutanásia seria uma conquista da humanidade. *(A special legislation allowing the practice of euthanasia would be an achievement of humanity.)*
**14**	Aquele que pensa saber a hora certa para alguém deixar de viver se julga Todo-Poderoso. *(Those who think knowing the right time for someone to stop living think they are Almighty.)*	Aquele que pensa saber a hora certa para alguém deixar de viver julga-se Todo-Poderoso. *(Those who think knowing the right time for someone to stop living think they are Almighty.)*
**15**	Se o doente não está mais lúcido e a família assumir a decisão, a eutanásia deve ser praticada. *(If the patient is no longer lucid and the family assumes the decision, euthanasia should be practiced.)*	Se o doente não está mais lúcido e a família assumir a decisão, a eutanásia deve ser praticada. *(If the patient is no longer lucid and the family assumes the decision, euthanasia should be practiced.)*
**16**	Por motivos vários não deve haver uma legislação especial permitindo a prática da eutanásia. *(For various reasons, there should be no special legislation allowing the practice of euthanasia.)*	Por motivos vários não deve haver uma legislação especial permitindo a prática da eutanásia. *(For various reasons, there should be no special legislation allowing the practice of euthanasia.)*
**17**	Há certas circunstâncias em que a pessoa tem todo o direito de decidir se quer ou não continuar esperando a morte. *(There are certain*	Há certas circunstâncias em que a pessoa tem todo o direito de decidir se quer ou não continuar à espera da morte. *(There are certain circumstances*

Item number	Original items of AAEs	Adjusted and final items of the aAAEs
	circumstances in which a person has every right to decide whether to continue waiting for death.)	*in which a person has every right to decide whether to continue waiting for death.)*
**18**	O direito de vida e o direito de morte escapam, sob qualquer circunstância, ao âmbito de decisão do Ser Humano. *(The right to life and the right to death escape, under any circumstances, the scope of decision of the Human Being.)*	O direito de vida e o direito de morte escapam, sob qualquer circunstância, ao âmbito de decisão do Ser Humano. *(The right to life and the right to death escape, under any circumstances, the scope of decision of the Human Being.)*
**19**	Ter direito à eutanásia é ter direito a uma morte digna. *(Having the right to euthanasia is having the right to a dignified death.)*	Ter direito à eutanásia é ter direito a uma morte digna. *(Having the right to euthanasia is having the right to a dignified death.)*
**20**	Praticar a eutanásia é matar por amor, mas não deixa de ser assassinato. *(Practicing euthanasia is killing for love, but it is still murder.)*	Praticar a eutanásia é matar por amor, mas não deixa de ser assassinato. *(Practicing euthanasia is killing for love, but it is still murder.)*
**21**	É um crime manter um ser humano em vida puramente vegetativa. *(Keeping a human being in a purely vegetative life is a crime.)*	É um crime manter um ser humano em vida puramente vegetativa. *(Keeping a human being in a purely vegetative life is a crime.)*
**22**	A eutanásia não se aplica a nenhum caso, pois cada doente reage de forma diferente a uma mesma doença. *(Euthanasia does not apply to any case, as each patient reacts differently to the same disease.)*	A eutanásia não se aplica a nenhum caso, pois cada doente reage de forma diferente a uma mesma doença. *(Euthanasia does not apply to any case, as each patient reacts differently to the same disease.)*
**23**	Uma legislação especial permitindo a prática da eutanásia deveria ser ampla, abrangendo o maior número de casos incuráveis e fatais. *(Special legislation allowing the practice of euthanasia should be broad, covering the greatest number of incurable and fatal cases.)*	Uma legislação especial permitindo a prática da eutanásia deveria ser ampla, abrangendo o maior número de casos incuráveis e fatais. *(Special legislation allowing the practice of euthanasia should be broad, covering the greatest number of incurable and fatal cases.)*
**24**	Quem autoriza a morte de um parente, na verdade, está querendo aliviar sua própria dor. *(Whoever authorizes the death of a relative truly want to alleviate their own pain.)*	Quem autoriza a morte de um parente, na verdade, quer aliviar a sua própria dor. *(Whoever authorize the death of a relative truly want to alleviate their own pain.)*
**25**	Se o desligamento da aparelhagem artificial não resolver, deve ser aplicada uma injeção que adiante a morte do condenado. *(If turning off the artificial equipment does not solve the problem, an injection must be applied to anticipate the death of the condemned patient.)*	Se desligar as máquinas de suporte artificial à vida não resolver, deve ser aplicada uma injeção que adiante a morte do condenado. *(If turning off the artificial life support machines does not solve the problem, an injection must be applied to anticipate the death of the condemned patient.)*
**26**	O sofrimento do doente incurável faz parte de sua missão na Terra, por isso a eutanásia não deve ser praticada. *(The suffering of the incurable patient is*	O sofrimento do doente incurável faz parte da sua missão na Terra, por isso a eutanásia não deve ser praticada. *(The suffering of the incurably ill is part*

Item number	Original items of AAEs	Adjusted and final items of the aAAEs
	part of their mission on Earth, so euthanasia should not be practiced.)	*of their mission on Earth, so euthanasia should not be practiced.)*

AAEs: attitude about euthanasia scale; aAAEs: adapted attitude about euthanasia scale
